# Supplementary material for: A Huluwa phosphorylation switch regulates embryonic axis induction
Source: Nat Commun. 2024 Nov 19;15:10028. doi: 10.1038/s41467-024-54450-4 (PMC11576741; doi:10.1038/s41467-024-54450-4)
Supplement: Supplementary file 1 — Supplementary Information [file 41467_2024_54450_MOESM1_ESM.pdf]

Supplementary Information for

A Huluwa phosphorylation switch regulates embryonic axis induction

Yao Li, Yun Yan, Bo Gong, Qianwen Zheng, Haiyan Zhou, Jiarui Sun, Mingpeng Li, Zhao Wang, Yaohui Li, Yunjing Wan, Weixi Chen, Shiqian Qi, Xianming Mo, Anming Meng, Bo Xiang, Jing Chen

*Correspondence author: jingchen@scu.edu.cn (J.C.)

This file includes:

1. **Supplementary Fig. 1- 11**
2. **Source data of Supplementary information：**

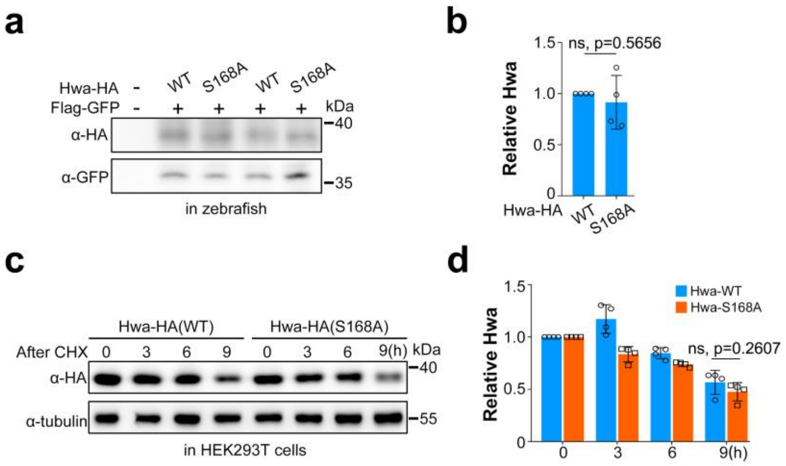

Supplementary Fig. 1. S168A point mutation does not affect the stability of Hwa. (**a**) Immunoblotting of Hwa-HA and Flag-GFP in zebrafish embryos injected with 200 pg WT or S168A mutant *hwa*-HA mRNA with 200 pg Flag-*gfp* mRNA at the 1-cell stage and harvested at 4 hpf. (**b**) Quantifications of relative Hwa protein levels in embryos treated as in (**a**), N=4. (**c**) Immunoblotting of Hwa in cells transfected with wild-type or S168A Hwa-HA plasmids, followed by treatment with cycloheximide (CHX; 100 μg/ml) and harvesting at the indicated time points. (**d**) Quantifications of relative Hwa protein levels treated as in (**c**), N=4. GFP (**a**) and α-tubulin (**c**) were used as references for quantification. (**b** & **d**) A two-tailed unpaired t test was performed and data were presented as mean ± SD. N, number of biological replicates; Significant differences are indicated by ns ≥ 0.05, *p < 0.05, **p < 0.01, ***p < 0.001, and ****p < 0.0001. Source data are provided at the end of the Supplementary Information file.

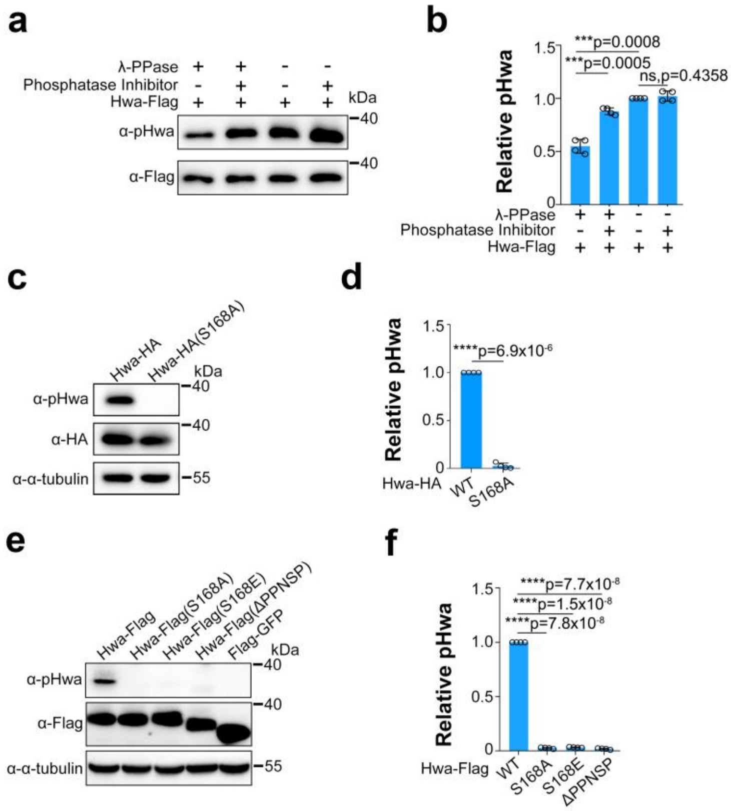

Supplementary Fig. 2. Verification of the specificity of the pHwa antibody.
(**a**) Immunoblotting of pHwa from samples treated with λ-PPase or λ-PPase & phosphatase inhibitor. (**b**) Quantifications of relative pHwa treated as in (**a**), N=4. (**c**) Immunoblotting of pHwa in HEK293T cells transfected with wild-type or S168A Hwa-HA mutant. (**d**) Quantifications of relative pHwa levels treated as in (**c**), N=4. (**e**) Immunoblotting of pHwa in HEK293T cells transfected with wild-type or mutants of Hwa-Flag. (**f**) Quantifications of relative pHwa treated as in (**e**), N=4. (**b**, **d** & **f**) A two-tailed unpaired t test was performed and data were presented as mean ± SD. N, number of biological replicates; Significant differences are indicated by ns ≥ 0.05, *$p < 0.05$, **$p < 0.01$, ***$p < 0.001$, and ****$p < 0.0001$. Source data are provided at the end of the Supplementary Information file.

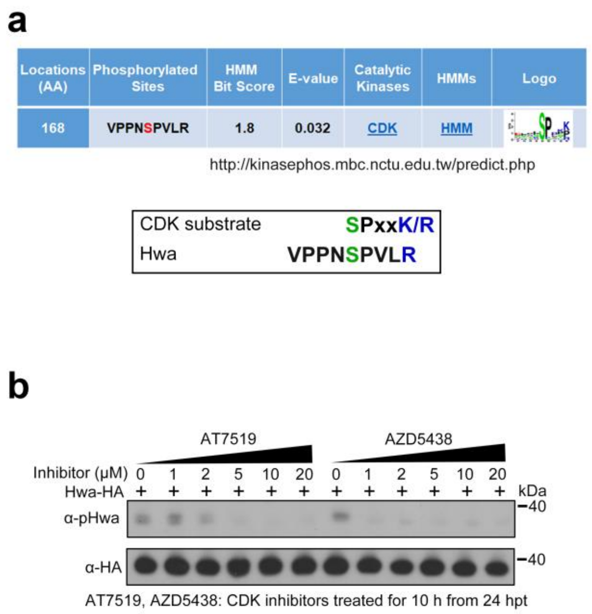

Supplementary Fig. 3. AT7519 and AZD5438 decreased pHwa in HEK293T cells.

(**a**) The upper panel shows the predicted protein kinase that may be involved in phosphorylation at Ser168; the lower panel indicates the consensus amino acid sequence of CDK substrates and that of Hwa. (**b**) Immunoblotting of pHwa and total Hwa-HA protein in HEK293T cells treated with different doses of CDK inhibitors (AT7519 and AZD5438) for 10 hours from 24 hours post transfection (hpt). Source data are provided at the end of the Supplementary Information file.

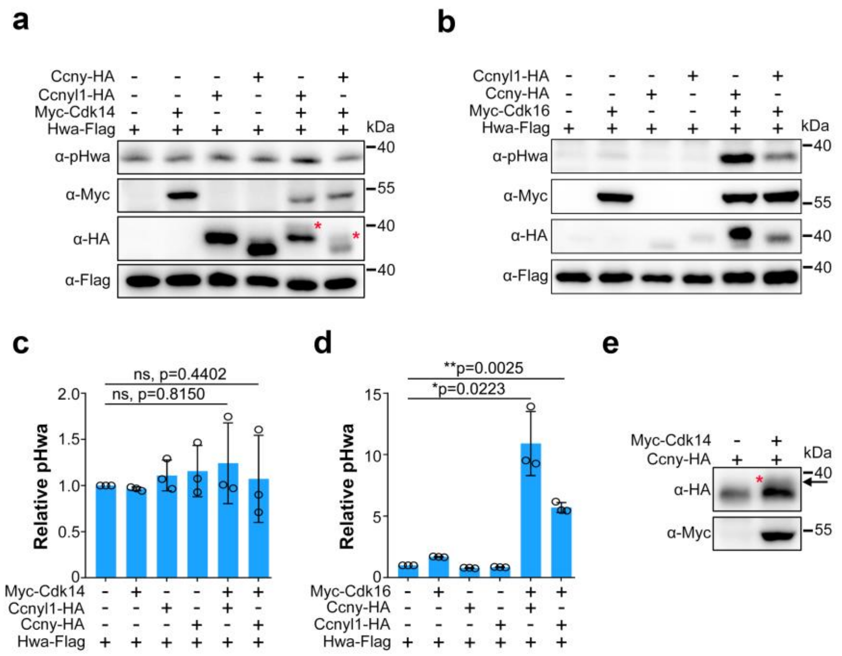

Supplementary Fig. 4. Cdk16, but not Cdk14, elevated pHwa with Ccny/Ccnyl1. (**a**) Immunoblotting of pHwa in HEK293T cells transfected with Hwa-Flag and Myc-Cdk14, in the absence or presence of Cyclin Y proteins (Ccny/Ccnyl1-HA). (**b**) Immunoblotting of pHwa in HEK293T cells transfected with Hwa-Flag and Myc-Cdk16, in the absence or presence of Cyclin Y (Ccny/Ccnyl1-HA). Total Hwa-Flag was applied as the reference. (**c**) and (**d**) Statistics of relative pHwa in (**a**) and (**b**), respectively, N=3. (**e**) Immunoblotting of phosphorylated Ccny (a migration-shift band indicated by the black arrow) in HEK293T cells transfected with Ccny-HA alone or cotransfected with Myc-Cdk14. The red star indicates the migration-shift band of phosphorylated Ccny/Ccnyl1 in (**a** & **e**). Total Hwa proteins (**a** & **b**) were used as references for quantification. (**c** & **d**) A two-tailed unpaired t test was performed and data were presented as mean ± SD. N, number of biological replicates; Significant differences are indicated by ns ≥ 0.05, *p < 0.05, **p < 0.01, ***p < 0.001, and ****p < 0.0001, with individual p-values shown. Source data are provided at the end of the Supplementary Information file.

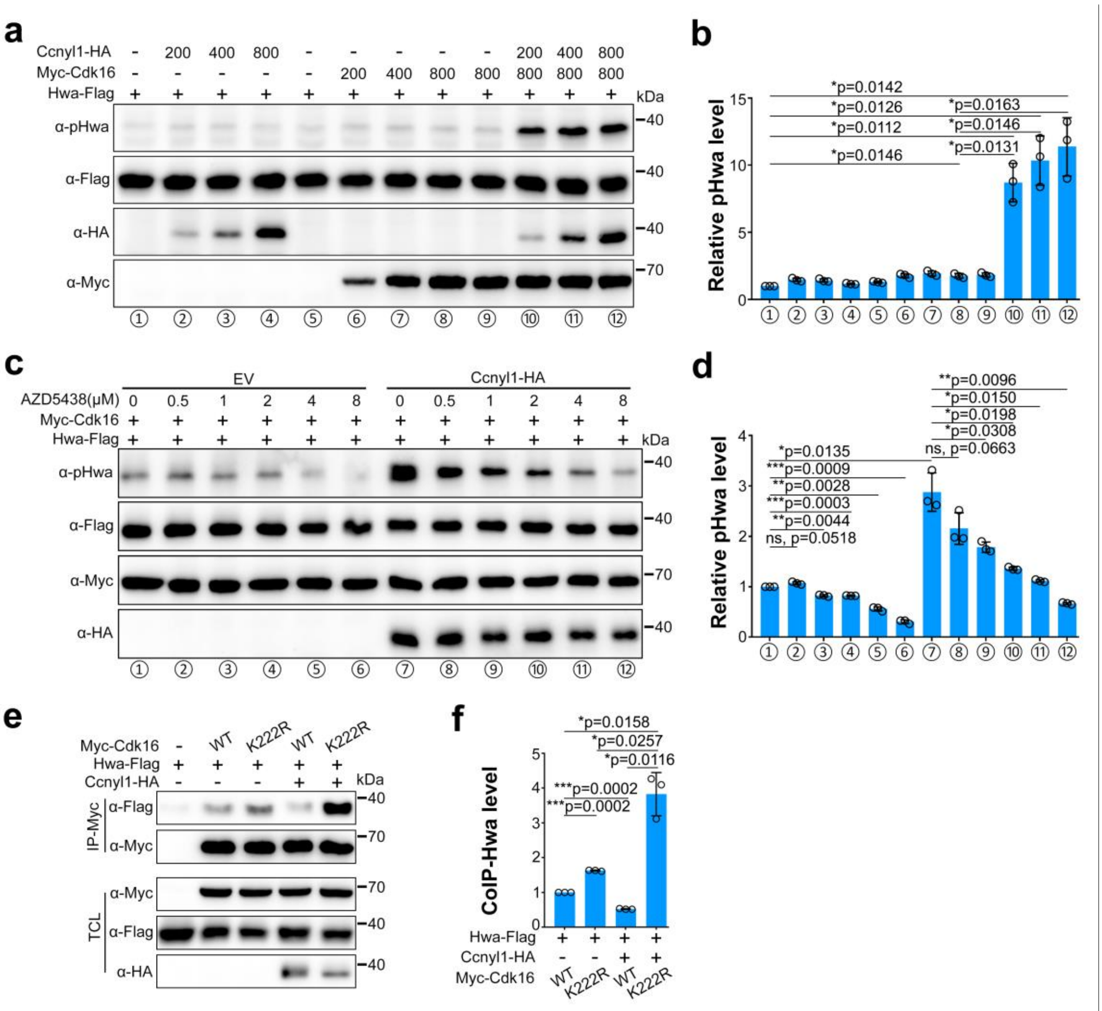

Supplementary Fig. 5. Cdk16 interacted with and phosphorylated Hwa.

(**a**) Immunoblotting of phosphorylated Hwa protein at Ser168 (pHwa) from HEK293T cells transfected with Hwa-Flag and Myc-Cdk16, in the absence or presence of Ccnyl1-HA. (**b**) Quantifications of relative pHwa levels in HEK293T cells treated as in (**a**), N=3. (**c**) Immunoblotting of pHwa from HEK293T cells cotransfected with Myc-Cdk16 and Ccnyl1-HA, followed by treatment with different doses of the CDK inhibitor AZD5438 (0-8 μM). (**d**) Quantifications of relative pHwa levels in HEK293T cells treated as in (**c**), N=3. (**e**) Coimmunoprecipitation of Hwa-Flag with wild-type and K222R mutant Myc-Cdk16 in the absence or presence of Ccnyl1-HA. The kinase-dead form Cdk16 showed a stronger interaction with Hwa in the presence of Ccnyl1-HA. (**f**) Quantifications of relative Co-IP Hwa in HEK293T cells treated as in (**e**), N=3. Total Hwa proteins (**a**, **c** & **e**) were used as references for quantification in immunoblotting. (**b**, **d** & **f**) A two-tailed unpaired t test was performed and data were presented as mean ± SD. N, number of biological replicates; Significant differences are indicated by ns ≥ 0.05, *p < 0.05, **p < 0.01, ***p < 0.001, and ****p < 0.0001, with individual p-value illustrated. Source data are provided at the end of the Supplementary Information file.

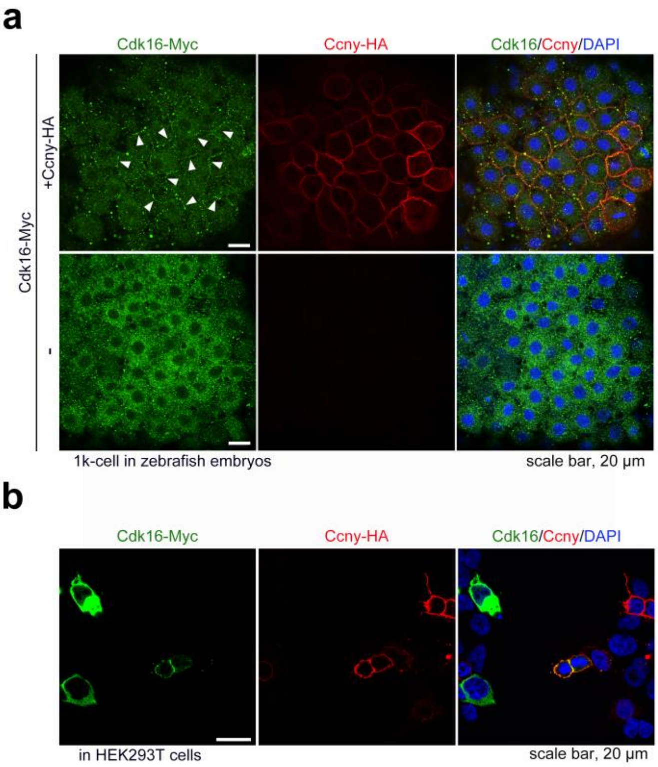

Supplementary Fig. 6. Cdk16 was recruited to plasm membrane by Ccny. (**a**) Immunofluorescence of Cdk16-Myc and Ccny-HA in zebrafish embryos at the 1k-cell stage, with arrowheads indicating the plasm membrane located Cdk16-Myc, N=3. (**b**) Immunofluorescence of Cdk16-Myc and Ccny-HA in HEK293T cells, N=3. Cells were stained with DAPI in blue; Scale bar, 20 μm. N, number of biological replicates.

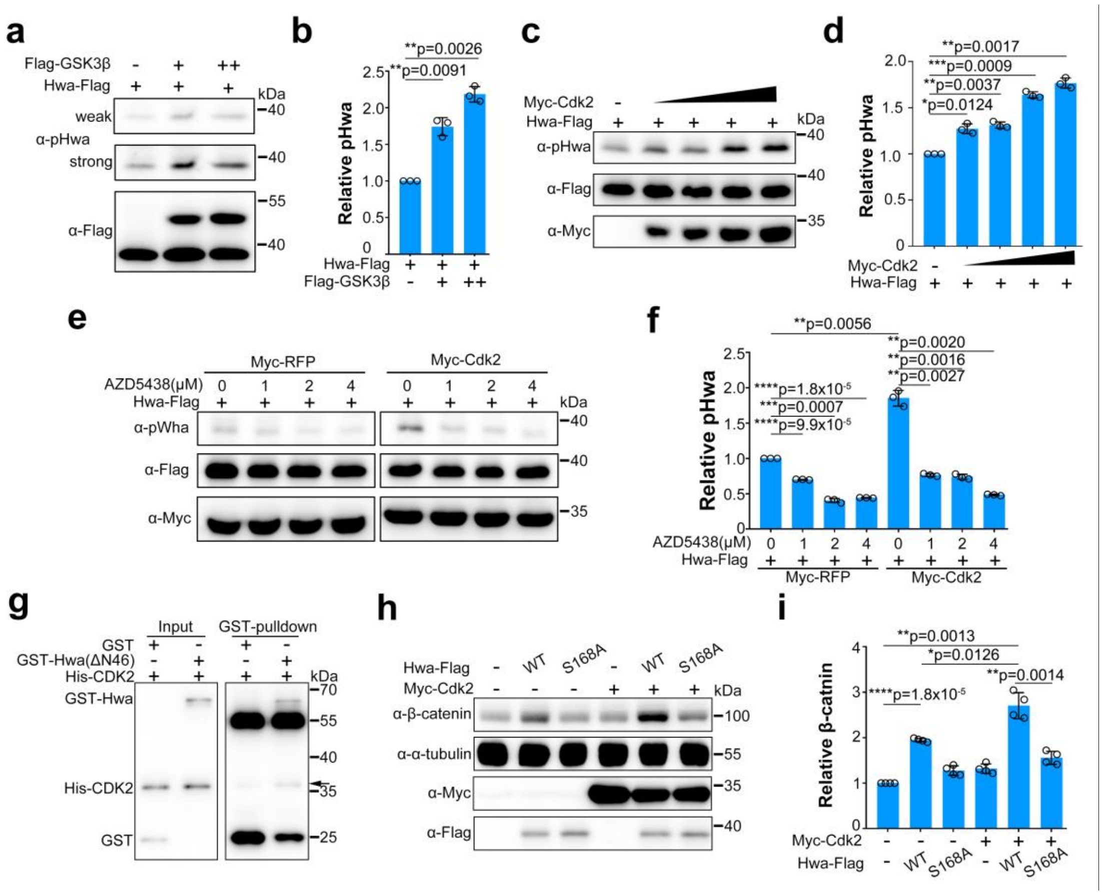

Supplementary Fig. 7. GSK3β and Cdk2 phosphorylates Hwa at Ser168.

(**a**) Immunoblotting of pHwa from HEK293T cells transfected with Hwa-Flag with or without Flag-tagged human GSK3β. (**b**) Quantifications of relative pHwa levels in HEK293T cells treated as in (**a**), N=3. (**c**) Immunoblotting of pHwa from HEK293T cells transfected with Hwa-Flag with different doses of Myc-Cdk2. (**d**) Quantifications of relative pHwa levels in HEK293T cells treated as in (**c**), N=3. (**e**) Immunoblotting of pHwa from HEK293T cells cotransfected Hwa-Flag with Myc-RFP or Myc-Cdk2, followed by treatment with different doses of the inhibitor AZD5438 (0-4 μM). (**f**) Quantifications of relative pHwa leves in HEK293T cells treated as in (**e**), N=3. (**g**) *in vitro* GST-pulldown assay of His-CDK2 with GST-Hwa(ΔN46) or GST, N=3. The arrow indicated the His-CDK2 protein. (**h**) Immunoblotting of cytosolic/active β-catenin in HEK293T cells transfected with Hwa-Flag (WT, S168A) alone or together with Myc-Cdk2. (**i**) Quantifications of relative pHwa levels in HEK293T cells treated as in (**h**), N=4. Total Hwa (**a**, **c** & **e**) and α-tubulin (**h**) proteins were used as references in immunoblotting. (**b**, **d**, **f** & **i**) A two-tailed unpaired t test was performed and data were presented as mean ± SD. N, number of biological replicates; Significant differences are indicated by ns ≥ 0.05, *p < 0.05, **p < 0.01, ***p < 0.001, and ****p < 0.0001, with individual p-value labeled. Source data are provided at the end of the Supplementary Information file.

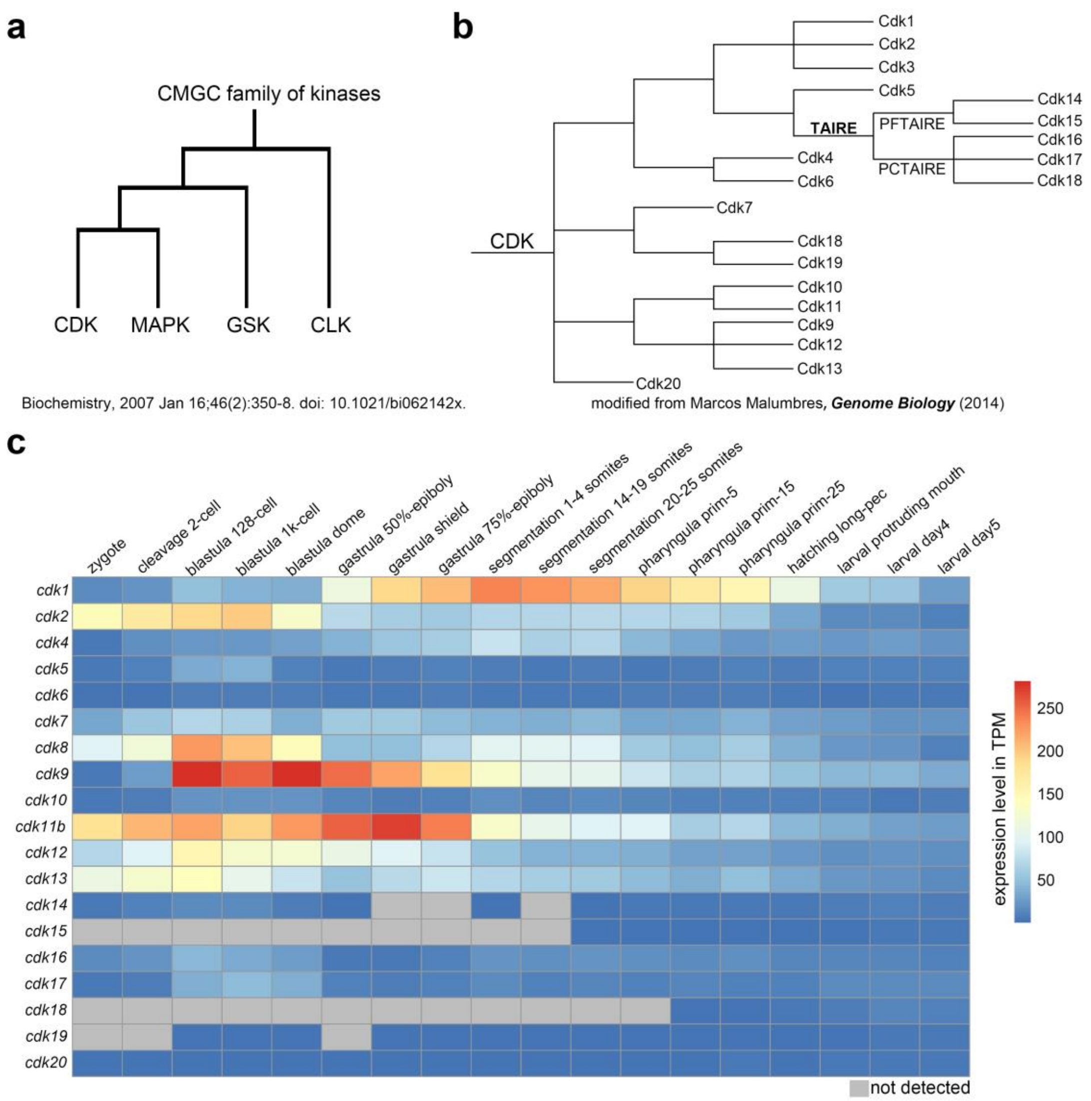

Supplementary Fig. 8. CMGC kinase family and expression profile of *cdk* genes in zebrafish.

(**a**) The classification of the CMGC kinase family. (**b**) Members of the CDK subfamily, derived from Marcos Malumbres, Genome Biology (2014). (**c**) Expression levels of different *cdk* genes during zebrafish early developmental stages were plotted with data from Expression Atlas. Expression levels are displayed in TPM (transcripts per million) with color coding, while gray color indicates “not detected”.

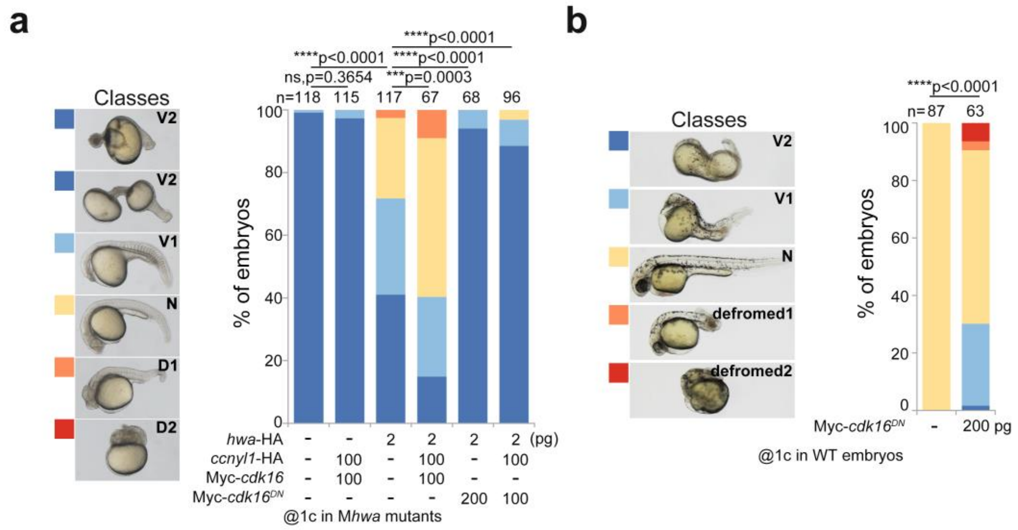

Supplementary Fig. 9. Cdk16^{DN} hindered the axis-inducing function of Hwa. (**a**) Rescue efficiency of M*hwa*^{tsu01sm} mutant embryos with a low dosage of *hwa* mRNA (2 pg) together with wild-type and the dominant negative form of Myc-*cdk16*^{DN} mRNA, N=2. Myc-*cdk16* mRNA coexpressed with *ccnyl1*-HA mRNA failed to rescue the M*hwa*$^{tsu01sm/tsu01sm}$ mutant phenotype without *hwa* mRNA. (**b**) Effects of overexpression of dominant negative *cdk16* (*cdk16*^{DN}) mRNAs in WT embryos: some embryos shown deformed phenotypes, N=2. V2<V1<N<D1<D2; V, ventralized; N, normal; D, dorsalized. (**a** & **b**) A two-tailed Fisher's exact test was performed to evaluate differences between treatments (all phenotypes were divided into two groups: Unchanged [Class I] and Changed [Clas II-V or Class II-IV]). N, number of biological replicates; n, total number of embryos in each treatment; Significant differences are indicated by ns ≥ 0.05, *$p < 0.05$, **$p < 0.01$, ***$p < 0.001$, and ****$p < 0.0001$. Source data are provided at the end of the Supplementary Information file.

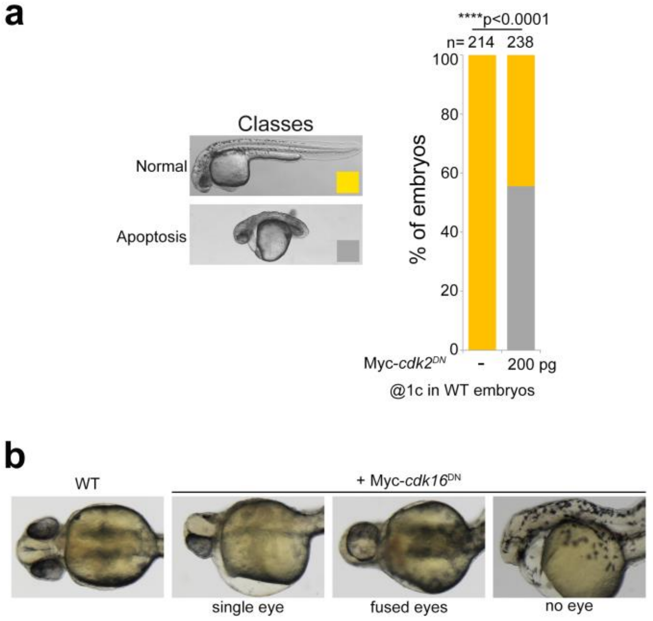

Supplementary Fig. 10. Phenotypes of Myc-*cdk2*^{DN} and Myc-*cdk16*^{DN} overexpression in WT embryos

(**a**) Effects of overexpression of 200 pg dominant negative *cdk2* (*cdk2^{DN}*) mRNA in wild-type embryos, N=4. (**b**) Injection of 200 pg Myc-*cdk16*^{DN} mRNA in WT embryos at the 1-cell stage resulted in some embryos with single-eye/fused-eyes/no-eye phenotypes, which was similar to that of blocking nodal signaling. (**a**) A two-tailed Fisher's exact test was performed to evaluate differences between treatments. N, number of biological replicates; n, total number of embryos in each treatment; Significant differences are indicated by ns $\geq$ 0.05, *p < 0.05, **p < 0.01, ***p < 0.001, and ****p < 0.0001. Source data are provided at the end of the Supplementary Information file.

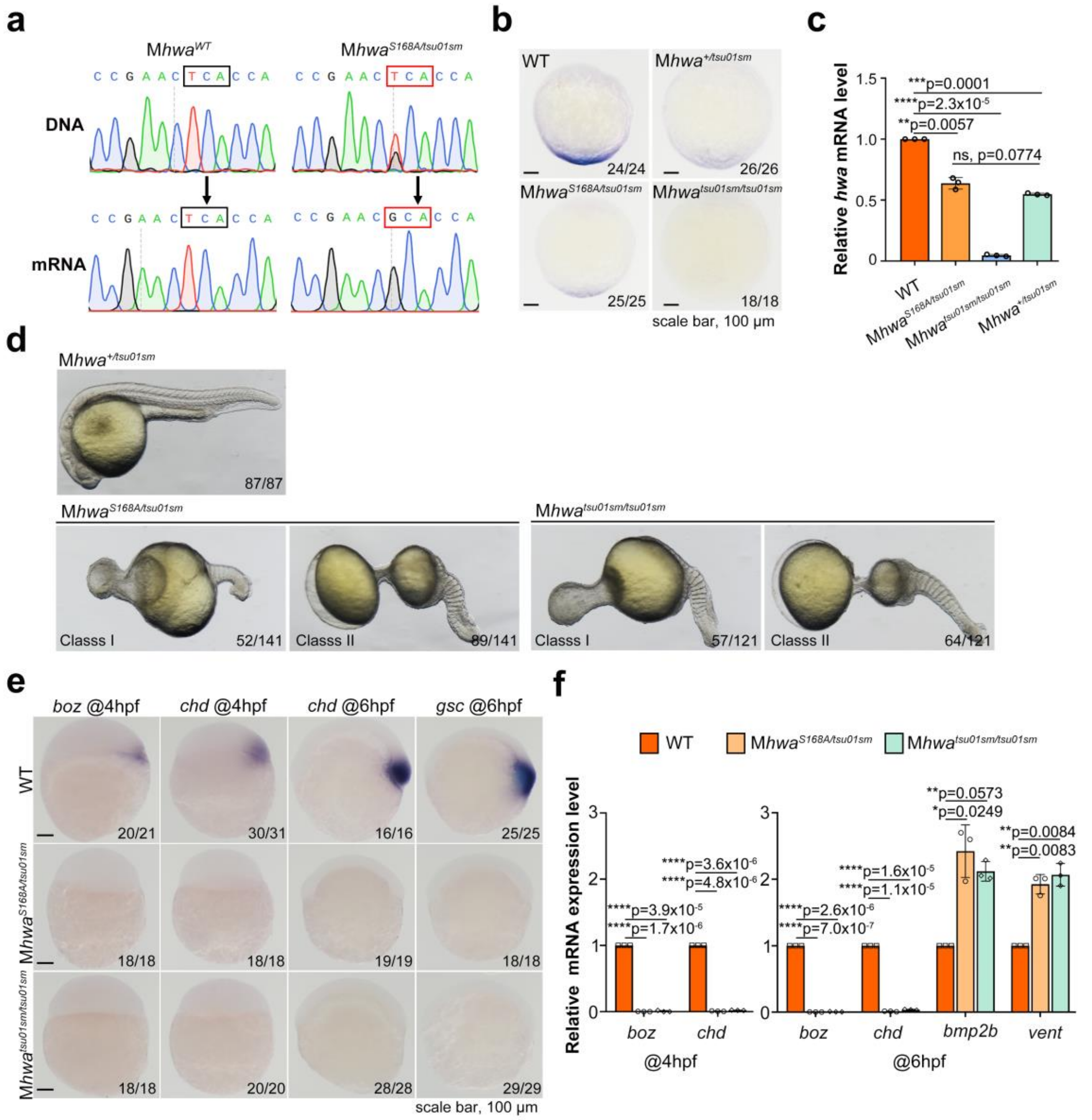

Supplementary Fig. 11. The endogenous function of the S168A mutation in zebrafish embryos.

(**a**) Sanger sequencing of WT and M*hwa*$^{S168A/tsu01sm}$ females (DNA in the upper panel) and derived embryos (mRNA in the lower panel) confirmed the successful construction of the *hwa*^{S168A} mutant line. WISH (**b**) and RT-qPCR (**c**) showed that the *hwa* mRNA levels were similar in M*hwa*$^{S168A/tsu01sm}$ and M*hwa*$^{+/tsu01sm}$ embryos, while the expression was almost absent in M*hwa*$^{tsu01sm/tsu01sm}$ embryos, N=3. (**d**) Phenotypes of M*hwa*$^{S168A/tsu01sm}$ embryos were similar to those from M*hwa*$^{tsu01sm /tsu01sm}$ mutants at 24 hpf and could be divided into two classes: both lacking an embryonic axis; while M*hwa*$^{+/tsu01sm}$ embryos were all normal. (**e**) Loss of expression of dorsal/organizer markers (*boz* and *chd* at 4 hpf; *chd* and *gsc* at 6 hpf) was detected by WISH in M*hwa*$^{S168A/tsu01sm}$ and M*hwa*$^{tsu01sm/tsu01sm}$ embryos. (**f**) Marker genes were quantified by RT-qPCR at 4 hpf and 6 hpf for WT, M*hwa*$^{S168A/tsu01sm}$ and M*hwa*$^{tsu01sm/tsu01sm}$ embryos, N=3. (**c & f**) A two-tailed unpaired t test was performed and data were presented as mean ± SD. N, number of biological replicates; Significant differences are indicated by ns ≥ 0.05, *$p < 0.05$, **$p < 0.01$, ***$p < 0.001$, and ****$p < 0.0001$, with individual p-

value labeled in each graph. Source data are provided at the end of the Supplementary Information file.

Source data of supplementary information：

Source data of Supplementary Fig. 1:

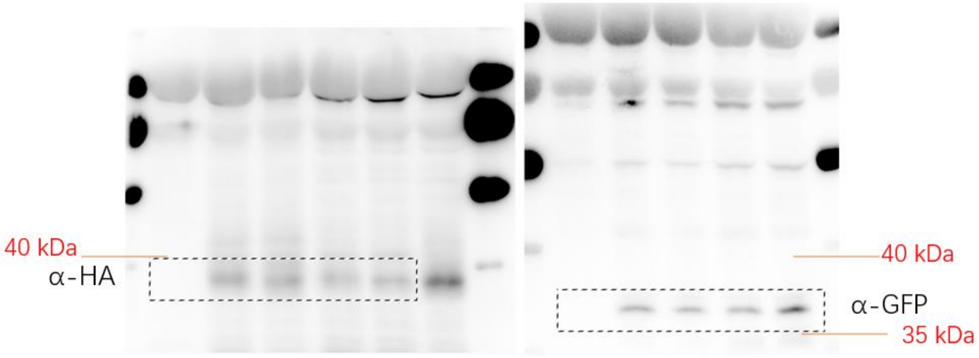

Supplementary Fig.1a, uncropped scans of all blots, detailed information has been labeled in the figure.

					Average	STDEV	p-value
WT-HA	1	1	1	1	1	0	
S168A-HA	1.254411	0.990007	0.687156	0.730356	0.915483	0.262572	0.565557

Supplementary Fig.1b, Specific values of quantifications of relative Hwa protein levels in embryos.

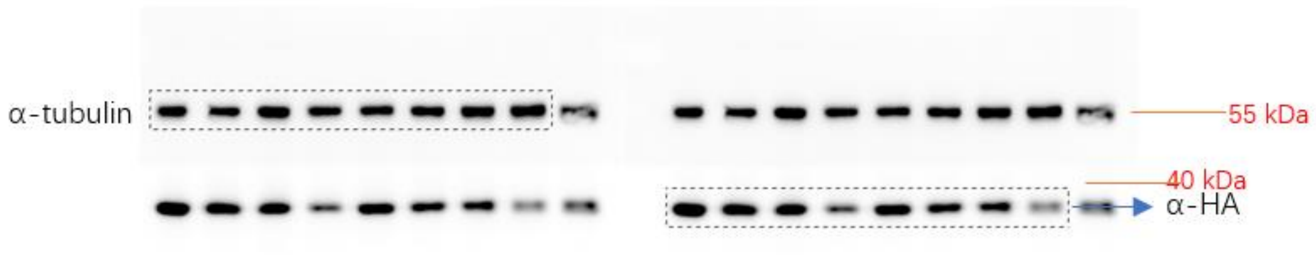

Supplementary Fig.1c, uncropped scans of all blots, detailed information has been labeled in the figure.

					Average	STDEV
WT-0	1	1	1	1	1	0
WT-3	1.304104	1.094027	1.019931	1.271177	1.17231	0.137225
WT-6	0.835441	0.881294	0.774472	0.889713	0.84523	0.052858
WT-9	0.396793	0.63434	0.600264	0.633806	0.566301	0.114124
S168A-0	1	1	1	1	1	0
S168A-3	0.729183	0.879277	0.847093	0.885779	0.835333	0.07276
S168A-6	0.740513	0.705717	0.754782	0.756497	0.739377	0.023556
S168A-9	0.347149	0.523938	0.49762	0.536679	0.476347	0.087653

Supplementary Fig.1d, Specific values of quantifications of relative Hwa protein levels.

Source data of Supplementary Fig. 2:

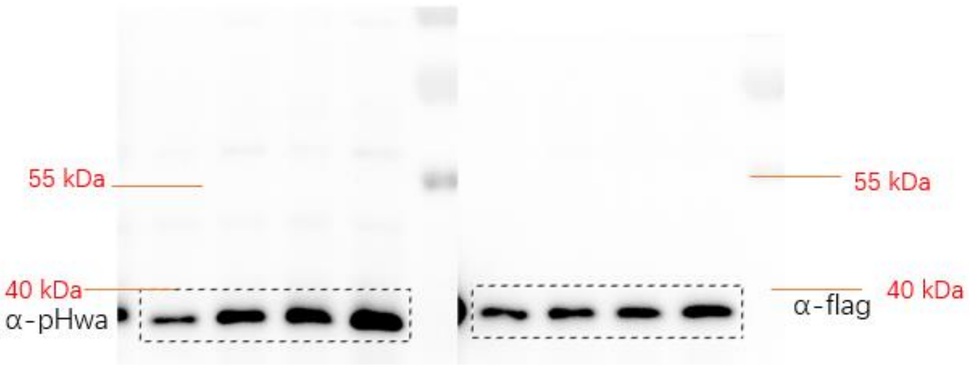

Supplementary Fig.2a, uncropped scans of all blots, detailed information has been labeled in the figure.

					average	STDEV
Ctr	1	1	1	1	1	0
Inhibitor	1.061319	0.980177	1.065272	0.979649	1.021604	0.048168
PPase	0.604031	0.476713	0.605391	0.511423	0.54939	0.065435
PPase+Inhibitor	0.906551	0.842963	0.905817	0.860212	0.878886	0.0323

Supplementary Fig.2b, Specific values of quantifications of relative pHwa.

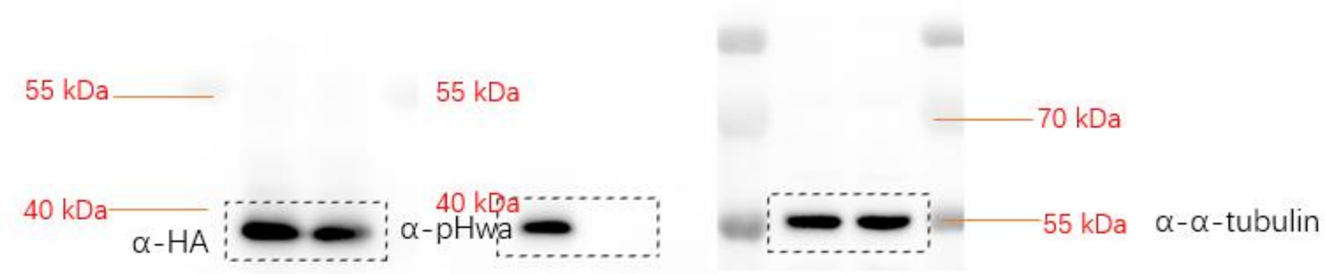

Supplementary Fig.2c, uncropped scans of all blots, detailed information has been labeled in the figure.

pHwa					Average	STDEV	p-Value
WT	1	1	1	1	1	0	
S168A	0.00816	0.022938	0.06629	0.003767	0.025289	0.028538	6.91E-06

Supplementary Fig.2d, Specific values of quantifications of relative pHwa levels.

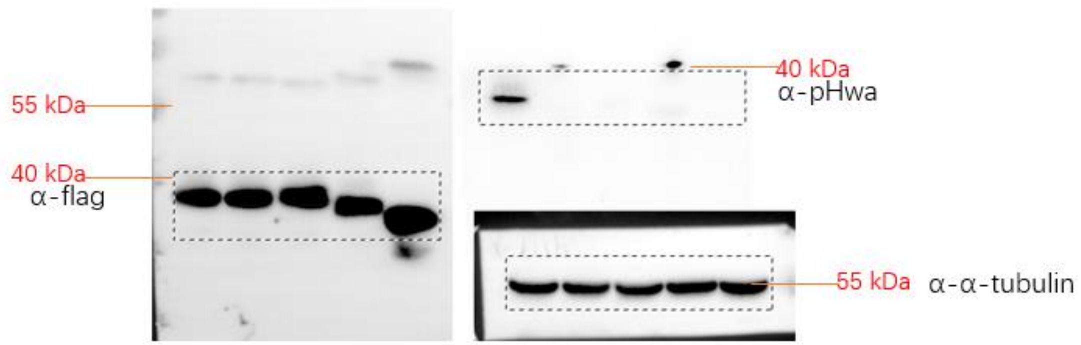

Supplementary Fig.2e, uncropped scans of all blots, detailed information has been labeled in the figure.

					Average	STDEV	P-Value
Flag-Hwa(WT)	1	1	1	1	1	0	
Flag-Hwa(S168A)	0.018082	0.030109	0.032583	0.025168	0.026485	0.006394	7.81E-08
Flag-Hwa(S168E)	0.029132	0.033351	0.037518	0.03077	0.032693	0.003656	1.49E-08
Flag-Hwa(dPPNSp)	0.019458	0.021089	0.009231	0.02395	0.018432	0.006409	7.67E-08

Supplementary Fig.2f, Specific values of quantifications of relative pHwa .

Source data of Supplementary Fig. 3:

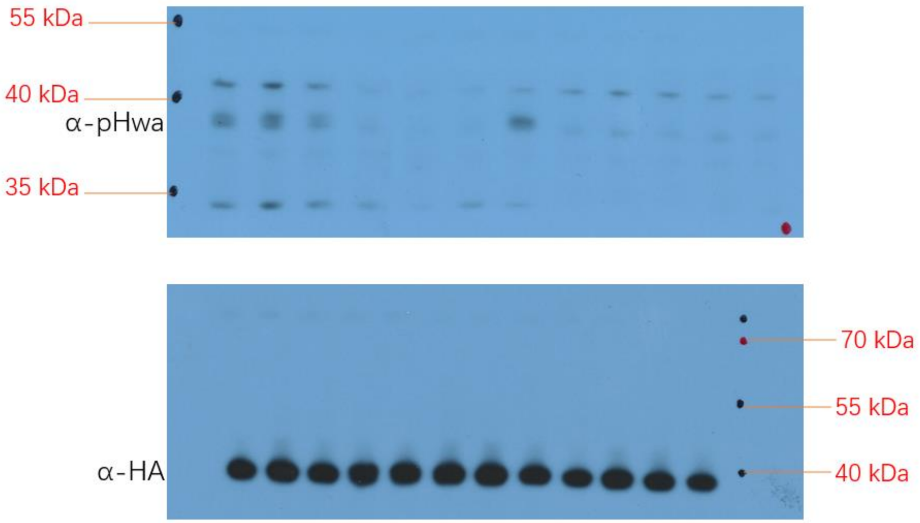

Supplementary Fig.3b, uncropped scans of all gels, detailed information has been labeled in the figure.

Source data of Supplementary Fig. 4:

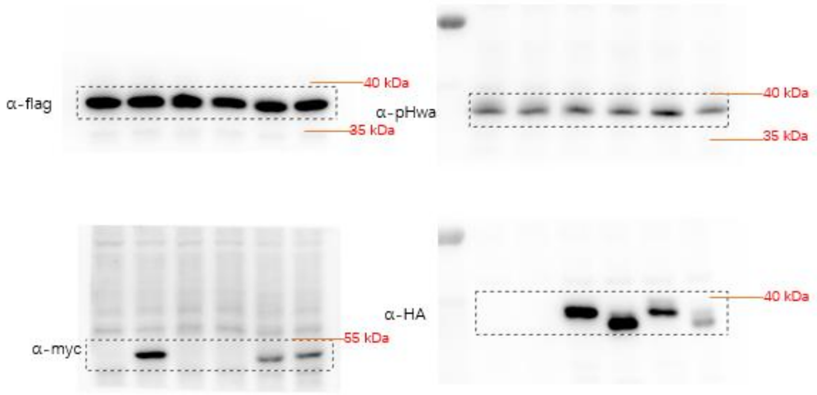

Supplementary Fig.4a, uncropped scans of all blots, detailed information has been labeled in the figure.

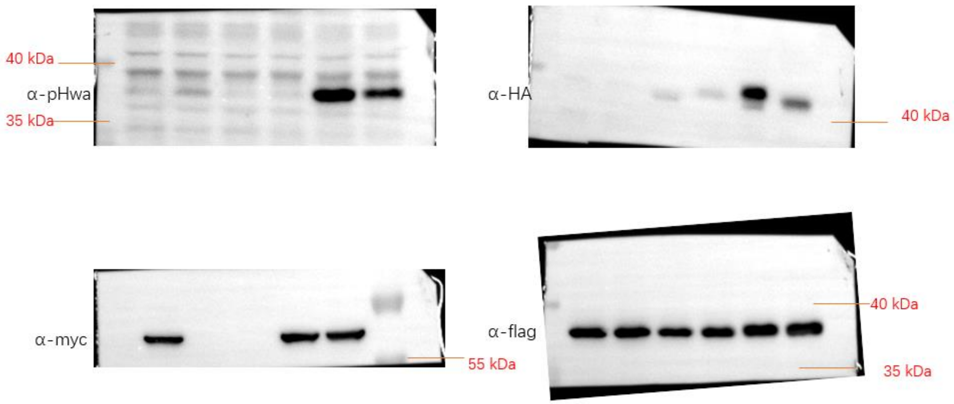

Supplementary Fig.4b, uncropped scans of all blots, detailed information has been labeled in the figure.

				Average	STDEV
EV	1	1	1	1	0
Cdk14	0.967145	0.941568	0.982109	0.963607	0.020501
Ccny	1.29237	0.964266	1.069514	1.108717	0.167528
Ccnyl1	1.467017	0.928605	1.074769	1.156797	0.278421
Cdk14+Ccny	1.746816	0.970754	1.007165	1.241578	0.437927
Cdk14+Ccnyl1	1.607466	0.900232	0.710281	1.07266	0.472794

Supplementary Fig.4c, Statistics of relative pHwa protein.

				Average	STDEV
EV	1	1	1	1	0
Cdk16	1.693286	1.6602	1.713051	1.688845	0.026704
Ccny	0.798929	0.753302	0.802192	0.784808	0.027333
Ccnyl1	0.836635	0.844341	0.885442	0.855473	0.026239
Cdk16+Ccny	13.90698	9.264641	9.534279	10.90197	2.60591
Cdk16+Ccnyl1	6.171502	5.482484	5.445777	5.699921	0.408814

Supplementary Fig.4d, Statistics of relative pHwa protein.

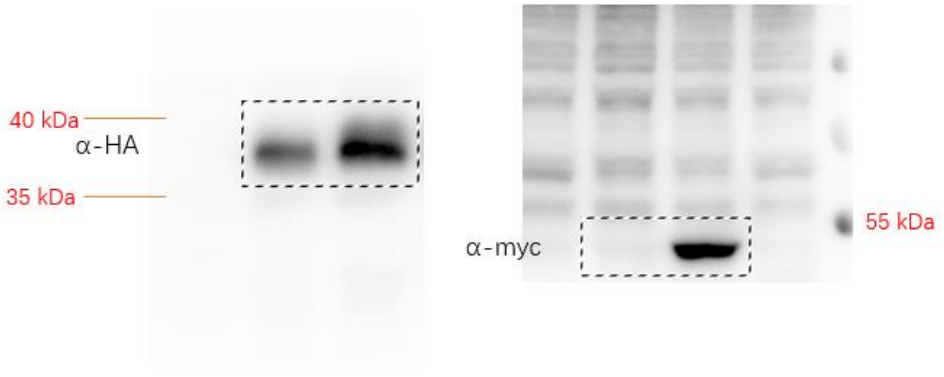

238
239 Supplementary Fig.4e, uncropped scans of all blots, detailed information has been labeled in the figure.
240

Source data of Supplementary Fig. 5:

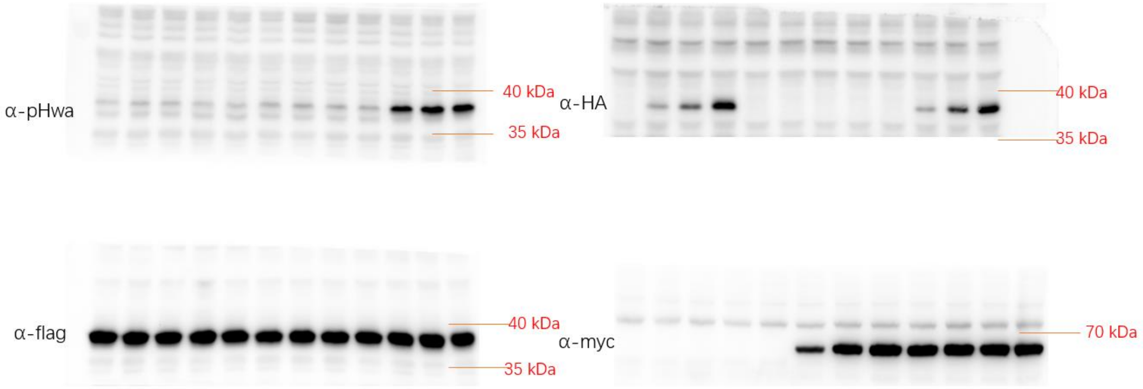

Supplementary Fig.5a, uncropped scans of all blots, detailed information has been labeled in the figure.

				Average	STDEV
Cdk16-1	1	1	1	1	0
Cdk16-2	1.427467	1.605504	1.363785	1.465585	0.125287
Cdk16-3	1.379244	1.551027	1.338199	1.422823	0.112908
Cdk16-4	1.142319	1.22073	1.132826	1.165292	0.048245
Cdk16-5	1.298487	1.354297	1.231817	1.294867	0.06132
Cdk16-6	1.819608	1.894624	1.615389	1.77654	0.144513
Cdk16-7	1.915496	2.143667	1.783137	1.947433	0.182375
Cdk16-8	1.730444	1.936633	1.618729	1.761935	0.161275
Cdk16-9	1.80057	2.008938	1.690429	1.833313	0.161759
Cdk16-10	8.79421	10.07848	7.237377	8.703356	1.422729

Cdk16-11	10.63168	12.01579	8.383637	10.3437	1.833123
Cdk16-12	11.87496	13.23049	8.995402	11.36695	2.162766

Supplementary Fig.5b, Specific values of quantifications of relative pHwa levels in HEK293T cells.

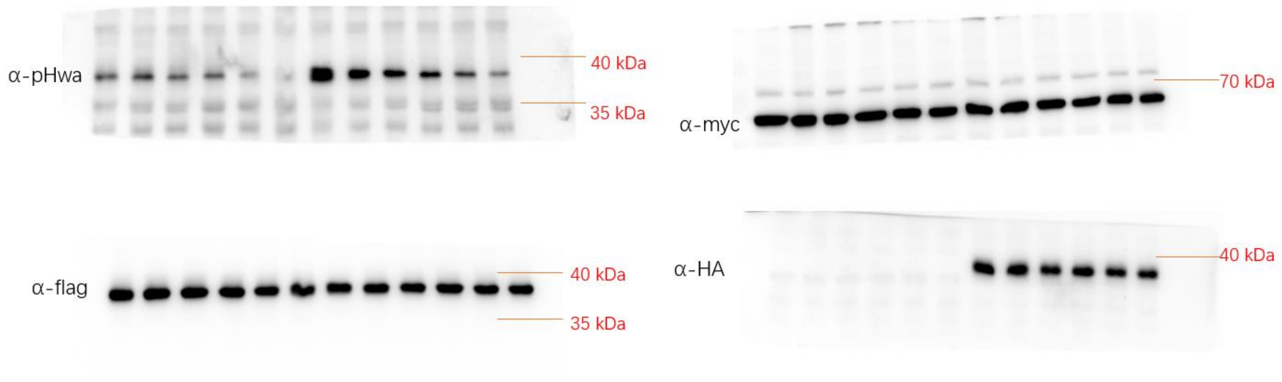

Supplementary Fig.5c, uncropped scans of all blots, detailed information has been labeled in the figure.

				Average	STDEV
EV_AZD 0	1	1	1	1	0
EV_AZD 0.5	1.043391	1.10078	1.066871	1.070347	0.028852
EV_AZD 1	0.801592	0.829972	0.840798	0.82412	0.020247
EV_AZD 2	0.817593	0.817212	0.827173	0.820659	0.005644
EV_AZD 4	0.577243	0.51176	0.585513	0.558172	0.040406
EV_AZD 8	0.325747	0.261532	0.325182	0.304153	0.036913
Cdk16+Ccnyl1_AZD 0	2.694781	3.315196	2.621445	2.877141	0.381135
Cdk16+Ccnyl1_AZD 0.5	1.981456	2.515808	1.961509	2.152924	0.314425

Cdk16+Ccnyl1_AZD 1	1.74355	1.900792	1.702903	1.782415	0.104512
Cdk16+Ccnyl1_AZD 2	1.342739	1.392122	1.336692	1.357185	0.030408
Cdk16+Ccnyl1_AZD 4	1.141979	1.096022	1.112227	1.116743	0.023309
Cdk16+Ccnyl1_AZD 8	0.685284	0.646207	0.670499	0.66733	0.01973

Supplementary Fig.5d, Specific values of quantifications of relative pHwa levels in HEK293T cells.

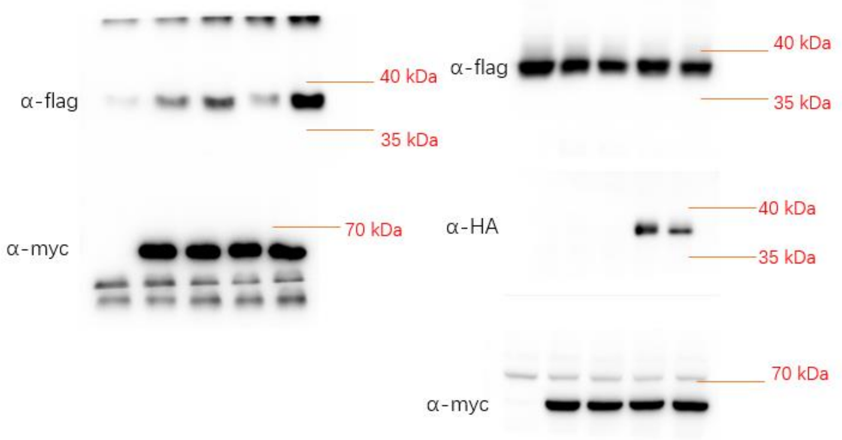

Supplementary Fig.5e, uncropped scans of all blots, detailed information has been labeled in the figure.

				Average	STDEV
CDK16_WT	1	1	1	1	0
CDK16_K222R	1.639776	1.608304	1.632062	1.626714	0.016404
CDK16_WT+Ccnyl1	0.517029	0.516846	0.535308	0.523061	0.010607
CDK16_K222R+Ccnyl1	3.112329	4.265601	4.095706	3.824545	0.62262

Supplementary Fig.5f, Specific values of quantifications of relative Co-IP Hwa in HEK293T cells.

Source data of Supplementary Fig. 7:

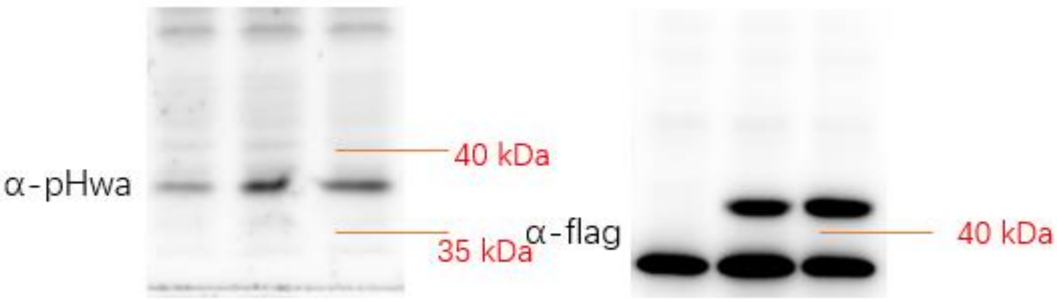

Supplementary Fig.7a, uncropped scans of all blots, detailed information has been labeled in the figure.

				Average	STDEV	P-Value
GSK3b-	1	1	1	1	0	
GSK3b+	1.825649	1.797905	1.599728	1.741094	0.12321	0.009088
GSK3b++	2.29869	2.091101	2.161732	2.183841	0.105546	0.002639

Supplementary Fig.7b, Specific values of quantifications of relative pHwa levels in HEK293T cells.

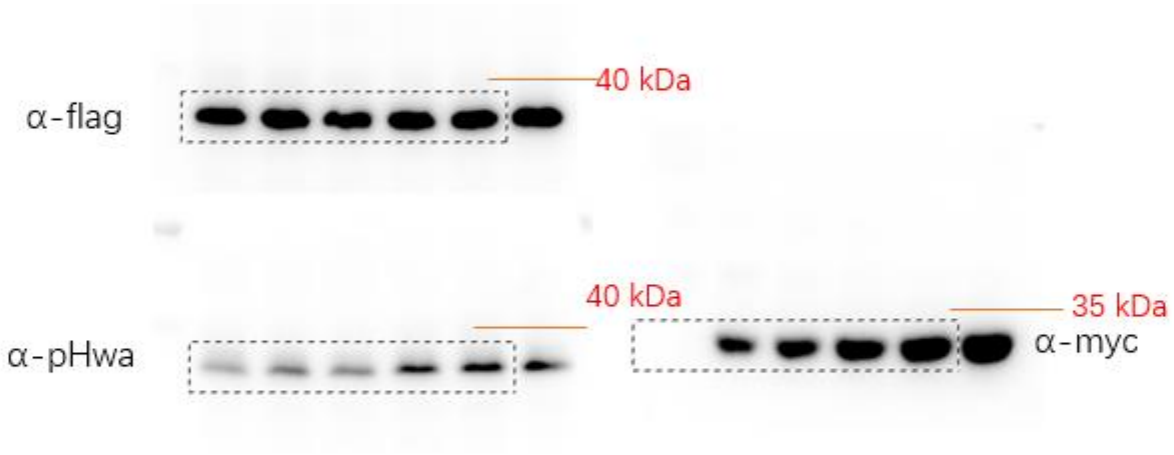

Supplementary Fig.7c, uncropped scans of all blots, detailed information has been labeled in the figure.

				Average	STDEV
Cdk2-0	1	1	1	1	0
Cdk2-1	1.260741	1.225759	1.329658	1.272053	0.052865
Cdk2-2	1.289344	1.296922	1.350254	1.312173	0.033196
Cdk2-3	1.614944	1.676971	1.624961	1.638959	0.033299
Cdk2-4	1.756869	1.826192	1.716371	1.766477	0.055537

Supplementary Fig.7d, Specific values of quantifications of relative pHwa levels in HEK293T cells.

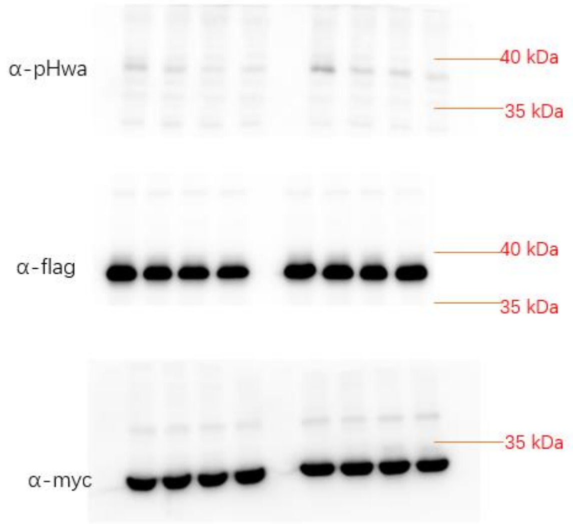

Supplementary Fig.7e, uncropped scans of all blots, detailed information has been labeled in the figure.

				Average	STDEV
EV_AZD-0	1	1	1	1	0
EV_AZD-1	0.703841	0.694373	0.70273	0.700315	0.005176
EV_AZD-2	0.369688	0.419241	0.414047	0.400992	0.027234
EV_AZD-4	0.435893	0.443512	0.442551	0.440652	0.004149
Cdk2_AZD-0	1.864454	1.734913	1.956351	1.851906	0.111251
Cdk2_AZD-1	0.763578	0.746115	0.785151	0.764948	0.019554
Cdk2_AZD-2	0.727468	0.710568	0.778845	0.73896	0.03556
Cdk2_AZD-4	0.472208	0.485426	0.492921	0.483518	0.010487

Supplementary Fig.7f, Specific values of quantifications of relative pHwa leves in HEK293T cells.

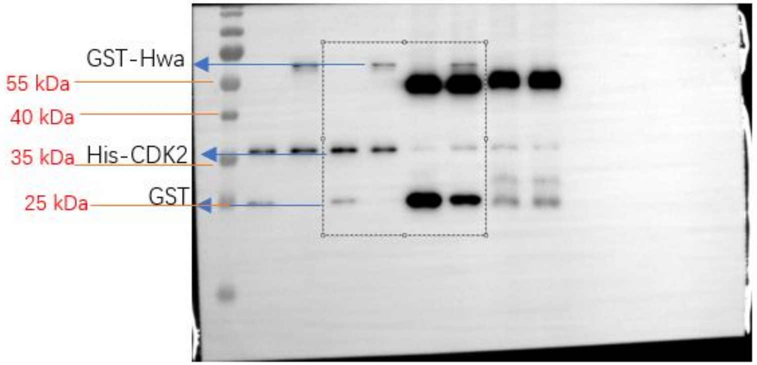

Supplementary Fig.7g, uncropped scans of blot, detailed information has been labeled in the figure.

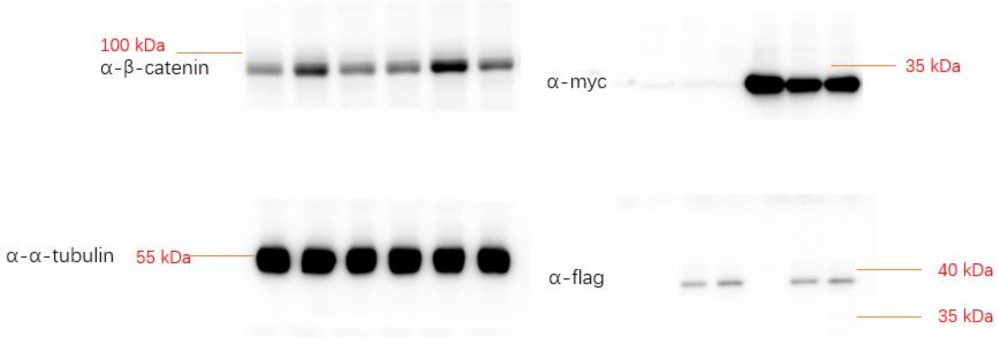

Supplementary Fig.7h, uncropped scans of all blots, detailed information has been labeled in the figure.

					Average	STDEV
EV	1	1	1	1	1	0
Hwa-WT	1.930155	1.96052	1.98722	1.899468	1.944341	0.037926
Hwa-S168A	1.398092	1.323582	1.195585	1.179132	1.274098	0.10489
EV+Cdk2	1.459175	1.307877	1.271426	1.216135	1.313653	0.104088
Hwa-WT+Cdk2	2.430845	3.043058	2.836223	2.499181	2.702327	0.288095
Hwa-S168A+Cdk2	1.721601	1.631977	1.409564	1.460514	1.555914	0.145782

Supplementary Fig.7i, Specific values of quantifications of relative pHwa levels in HEK293T cells.

Source data of Supplementary Fig. 9:

	V2	V1	N	D1	D2	
Sum	ClassI	ClassII	ClassIII	ClassIV	ClassV	Total
Mhwa	117	1	0	0	0	118
GFP 2pg+cdk16+ccnyl1 100+100pg	112	3	0	0	0	115
hwa-HA 2pg+GFP 200pg	48	36	30	3	0	117
hwa-HA 2pg+cdk16+ccnyl1 100+100pg	10	17	34	6	0	67
hwa-HA 2pg+cdk16-DN 200pg	64	4	0	0	0	68
hwa-HA 2pg+cdk16-DN-ccnyl2 100+100pg	85	8	3	0	0	96

Supplementary Fig.9a, Specific statistics in Fig.9a, numbers represent the number of zebrafish embryos.

WT	V2	V1	N	deformed1	deformed2	
	ClassI	ClassII	ClassIII	ClassIV	ClassV	Total
AB	0	0	87	0	0	87
AB+cdk16-DN200	1	18	38	2	4	63

Supplementary Fig.9b, specific statistics in Fig.9b, numbers represent the number of zebrafish embryos.

Source data of Supplementary Fig. 10:

WT embryos			
Summary	Normal	Apoptosis	Total
uninjection	214	0	214
cdk2 DN	121	117	238

Supplementary Fig.10a, specific statistics in Fig.10a, numbers represent the number of zebrafish embryos.

Source data of Supplementary Fig. 11:

				Average	STDEV
WT	1	1	1	1	0
S168A/tsu01sm	0.64551	0.586957	0.681206	0.637891	0.047584
tsu01sm/tsu01sm	0.054354	0.045769	0.03851	0.046211	0.007931
wt/tsu01sm	0.540034	0.546307	0.558175	0.548172	0.009213

Supplementary Fig.11c, specific statistics of relative hwa mRNA levels

4 hpf	WT			S168A/tsu01sm			tsu01sm/tsu01sm		
boz	1	1	1	0.003911	5.35E-05	4.94E-05	0.019432237	0.000209	0.001636
chd	1	1	1	0.0066	0.005645	0.01257	0.018297691	0.02469	0.020903
6 hpf	WT			S168A/tsu01sm			tsu01sm/tsu01sm		
chd	1	1	1	0	0.002796	0.000716	0.003762596	0.002397	0.007758
gsc	1	1	1	0.004144	0.015092	0.012061	0.040408956	0.035526	0.027256
bmp	1	1	1	1.969831	2.701364	2.599216	2.015618358	2.287804	2.053604
vent	1	1	1	1.756268	2.009843	2.012396	1.902025732	2.056002	2.241871

Supplementary Fig.11f, relative mRNA expression levels of the organizer-specific genes (boz, chd, gsc, bmp2b, and vent) at different stages.
