# Supplementary material for: A Huluwa phosphorylation switch regulates embryonic axis induction
Source: Nat Commun. 2024 Nov 19;15:10028. doi: 10.1038/s41467-024-54450-4 (PMC11576741; doi:10.1038/s41467-024-54450-4)
Supplement: Supplementary file 3 — Reporting Summary [file 41467_2024_54450_MOESM3_ESM.pdf]

nature portfolio

Corresponding author(s): COMMS-24-06806;Jing Chen

Last updated by author(s): Oct 30, 2024



# Reporting Summary

Nature Portfolio wishes to improve the reproducibility of the work that we publish. This form provides structure for consistency and transparency in reporting. For further information on Nature Portfolio policies, see our Editorial Policies and the Editorial Policy Checklist.

## Statistics

For all statistical analyses, confirm that the following items are present in the figure legend, table legend, main text, or Methods section.

| n/a | Confirmed | |
|---|---|---|
| ☐ | ☒ | The exact sample size (*n*) for each experimental group/condition, given as a discrete number and unit of measurement |
| ☐ | ☒ | A statement on whether measurements were taken from distinct samples or whether the same sample was measured repeatedly |
| ☐ | ☒ | The statistical test(s) used AND whether they are one- or two-sided<br>*Only common tests should be described solely by name; describe more complex techniques in the Methods section.* |
| ☐ | ☒ | A description of all covariates tested |
| ☐ | ☒ | A description of any assumptions or corrections, such as tests of normality and adjustment for multiple comparisons |
| ☐ | ☒ | A full description of the statistical parameters including central tendency (e.g. means) or other basic estimates (e.g. regression coefficient) AND variation (e.g. standard deviation) or associated estimates of uncertainty (e.g. confidence intervals) |
| ☒ | ☐ | For null hypothesis testing, the test statistic (e.g. *F*, *t*, *r*) with confidence intervals, effect sizes, degrees of freedom and *P* value noted<br>*Give P values as exact values whenever suitable.* |
| ☒ | ☐ | For Bayesian analysis, information on the choice of priors and Markov chain Monte Carlo settings |
| ☐ | ☒ | For hierarchical and complex designs, identification of the appropriate level for tests and full reporting of outcomes |
| ☒ | ☐ | Estimates of effect sizes (e.g. Cohen's *d*, Pearson's *r*), indicating how they were calculated |

*Our web collection on statistics for biologists contains articles on many of the points above.*

## Software and code

Policy information about availability of computer code

| | |
|---|---|
| Data collection | Silico kinase prediction on the website (http://kinasephos.mbc.nctu.edu.tw/predict.php) was used to identify the kinase responsible for phosphorylating Hwa at Ser168. |
| Data analysis | All immunoblotting and RT-qPCR experiments were repeated at least three times (N ≥ 3) with the individual data points shown in the bar graphs plotted by GraphPad Prism 8. Data for statistical analysis are presented as the mean ± S.D. The significance of differences between treatments were analyzed using a two-tailed unpaired t test without special mentions (Fig. 2c-d, 2g, 4g-j, 4n-p, 5b, 5d, 5g, 5i, 6c, 7d and Supplementary Fig.1b, 1d, 2b, 2d, 2f, 4c-d, 5b, 5d, 5f, 7b, 7d, 7f, 7i, 11c, 11f). For microinjection and treatment of embryos, results from repeated experiments (N ≥ 2) were summed to show the total number of each treatment (n). The effects of different treatments were compared using Fisher's exact test in GraphPad Prism (all phenotypes were divided into two groups: Unchanged [Class I] and Changed [Class II-V or Class II-IV]) (Fig. 1c, 1f, 2a, 5e-f, 5h, 5j, 6e-g, and Supplementary Fig. 9a-b, 10a ). Significance levels are indicated by ns ≥ 0.05, *p < 0.05, **p < 0.01, ***p < 0.001, and ****p < 0.0001, with the p-value shown in the individual figures. The sample size (n) and number of replications (N) of the experiment are described in the legend of each figure. |

For manuscripts utilizing custom algorithms or software that are central to the research but not yet described in published literature, software must be made available to editors and reviewers. We strongly encourage code deposition in a community repository (e.g. GitHub). See the Nature Portfolio guidelines for submitting code & software for further information.

## Data

Policy information about availability of data

All manuscripts must include a data availability statement. This statement should provide the following information, where applicable:

- Accession codes, unique identifiers, or web links for publicly available datasets
- A description of any restrictions on data availability
- For clinical datasets or third party data, please ensure that the statement adheres to our policy

All data are included in this manuscript, and source data are provided with this paper.

## Research involving human participants, their data, or biological material

Policy information about studies with human participants or human data. See also policy information about sex, gender (identity/presentation), and sexual orientation and race, ethnicity and racism.

| | |
|---|---|
| Reporting on sex and gender | n/a |
| Reporting on race, ethnicity, or other socially relevant groupings | n/a |
| Population characteristics | n/a |
| Recruitment | n/a |
| Ethics oversight | n/a |

Note that full information on the approval of the study protocol must also be provided in the manuscript.

# Field-specific reporting

Please select the one below that is the best fit for your research. If you are not sure, read the appropriate sections before making your selection.

☒ Life sciences ☐ Behavioural & social sciences ☐ Ecological, evolutionary & environmental sciences

For a reference copy of the document with all sections, see nature.com/documents/nr-reporting-summary-flat.pdf

# Life sciences study design

All studies must disclose on these points even when the disclosure is negative.

| | |
|---|---|
| Sample size | Sample sizes were determined with reference of similar experiments in previously published articles. We used 50 to over a hundred embryos for rescue experiments and subsequent phenotypic statistics. |
| Data exclusions | The malformed embryos with unrelated phenotypes due to natural and injection factors are excluded. |
| Replication | Each of the experiments are repeated at least twice and got consistent results for the trends. |
| Randomization | Before the samples or embryos were grouped for processing, they were confluent and then randomly grouped forprocessing. |
| Blinding | We blinded the data analysis and processing of the results by removing the group names and then analyzing them, and then adding the group names after the analysis was completed. |

# Reporting for specific materials, systems and methods

We require information from authors about some types of materials, experimental systems and methods used in many studies. Here, indicate whether each material, system or method listed is relevant to your study. If you are not sure if a list item applies to your research, read the appropriate section before selecting a response.

## Materials & experimental systems

| n/a | Involved in the study |
|---|---|
| ☐ | ☒ Antibodies |
| ☐ | ☒ Eukaryotic cell lines |
| ☒ | ☐ Palaeontology and archaeology |
| ☐ | ☒ Animals and other organisms |
| ☒ | ☐ Clinical data |
| ☒ | ☐ Dual use research of concern |
| ☒ | ☐ Plants |

## Methods

| n/a | Involved in the study |
|---|---|
| ☒ | ☐ ChIP-seq |
| ☒ | ☐ Flow cytometry |
| ☒ | ☐ MRI-based neuroimaging |

# Antibodies

Antibodies used

anti-Flag (F1804, Sigma, 1:1000), anti-Myc 444 (AE010, ABclonal, 1:1000; sc-40, Santa Cruz; proteintech, 60003, 1:5000), anti-HA antibody (sc-7392, Santa Cruz; AE008, 445 ABclonal; proteintech, 51064, 1:3000), anti-β-catenin (8480S, CST, 1:3000), anti-α-tubulin (T6199, Sigma; AC008, 446 ABclonal, 1:2000), anti-β-actin (AC026, ABclonal, 1:4000), anti-His (AE003, ABclonal, 1:5000), anti-GST 447 (AF5063B, Beyotime Biotechnology,1:2000), Anti-pHwa(Ser168) 454 (antiserum/antibody, produced by Shanghai Genomics in rabbits with synthetic 455 phosphorylated peptides (VNTVPPN(p)SPVLR), purified by HUABIO, Hangzhou,1:3000).

Validation

Validated for effectiveness and specificity in both cellular and embryonic samples.

# Eukaryotic cell lines

Policy information about cell lines and Sex and Gender in Research

Cell line source(s)

The HEK293T cell line was obtained from Prof. Kui Wang of Sichuan University.

Authentication

Cells were authenticated by the supplier using STR analysis.

Mycoplasma contamination

Routine test for mycoplasma was conducted by direct Hoechst  dyes staining of mycoplasma DNA or PCR test.

Commonly misidentified lines
(See ICLAC register)

No commonly misidentified lines were used.

# Animals and other research organisms

Policy information about studies involving animals; ARRIVE guidelines recommended for reporting animal research, and Sex and Gender in Research

Laboratory animals

The zebrafish was used as the experimental animal and was obtained from the China Zebrafish Resource Center.

Wild animals

n/a

Reporting on sex

n/a

Field-collected samples

n/a

Ethics oversight

Embryos were raised at 28.5 °C and staged as previously described in the article by Kimmel et al. Fish maintenance and breeding followed the institutional animal care and use committee (ACUC protocol, with approval by the Animal Care and Use Committee of West China Hospital. Sichuan University (NO,20220422003).

Note that full information on the approval of the study protocol must also be provided in the manuscript.

# Plants

Seed stocks

n/a

Novel plant genotypes

n/a

Authentication

n/a
